# Supplementary material for: Importance of broken geometric symmetry of single-atom Pt sites for efficient electrocatalysis
Source: Nat Commun. 2023 Jun 3;14:3233. doi: 10.1038/s41467-023-38964-x (PMC10239452; doi:10.1038/s41467-023-38964-x)
Supplement: Supplementary file 1 — Supplementary Information [file 41467_2023_38964_MOESM1_ESM.pdf]

Supplementary Information

Importance of Broken Geometric Symmetry of Single-Atom Pt Sites for Efficient Electrocatalysis

Junsic Cho[1,†], Taejung Lim[2,†], Haesol Kim[1,†], Ling Meng[3], Jinjong Kim[2,4], Seunghoon Lee[1], Jong Hoon Lee[5], Gwan Yeong Jung[6], Kug-Seung Lee[7], Francesc Viñes[3], Francesc Illas[3], Kai S. Exner[8,*], Sang Hoon Joo[2,4,*] & Chang Hyuck Choi[1,9,*]

[1]Department of Chemistry, Pohang University of Science and Technology (POSTECH), Pohang 37673, Republic of Korea.

[2]Department of Chemistry, Ulsan National Institute of Science and Technology (UNIST), Ulsan 44919, Republic of Korea.

[3]Departament de Ciència de Materials i Química Física & Institut de Química Teòrica i Computacional (IQTCUB), Universitat de Barcelona, c/ Martí i Franquès 1-11, 08028 Barcelona, Spain.

[4]Department of Chemistry, Seoul National University, Seoul 08826, Republic of Korea.

[5]UNIST Central Research Facilities (UCRF), Ulsan National Institute of Science and Technology (UNIST), Ulsan 44919, Republic of Korea.

[6]School of Energy and Chemical Engineering, Ulsan National Institute of Science and Technology (UNIST), Ulsan 44919, Republic of Korea.

[7]Beamline Department, Pohang Accelerator Laboratory, Pohang University of Science and Technology (POSTECH), Pohang 37673, Republic of Korea.

[8]Faculty of Chemistry, Theoretical Inorganic Chemistry, University of Duisburg-Essen, 45141 Essen, Germany; Cluster of Excellence RESOLV, 44801 Bochum, Germany; Center for Nanointegration Duisburg-Essen (CENIDE), 47057 Duisburg, Germany.

[9]Institute for Convergence Research and Education in Advanced Technology (I-CREATE), Yonsei University, Seoul 03722, Republic of Korea.

†These authors contributed equally: Junsic Cho, Taejung Lim, and Haesol Kim

*Corresponding authors: (K.S.E) kai.exner@uni-due.de; (S.H.J) shjoo1@snu.ac.kr; (C.H.C) chchoi@postech.ac.kr

Supplementary Note 1

Physical characterization of Pt_1(X)/CNTs

High-angle annular dark-field scanning transmission electron microscopy (HAADF-STEM) analysis was performed to identify the atomic distribution of the Pt species on Pt_1(X)/CNT, where the subscript '1' indicates the atomic isolation of Pt, and the number in parenthesis indicates the Pt content in wt.%. The results showed a uniform distribution of atomically dispersed Pt species without appreciable amounts of Pt clusters or nanoparticles (Supplementary Fig. 1). The Pt contents of Pt_1(3)/CNT, Pt_1(1)/CNT, and Pt_1(0.15)/CNT were 3, 1, and 0.15 wt.%, respectively, confirmed by inductively coupled plasma-optical emission spectroscopy (ICP-OES). In the X-ray diffraction (XRD) spectrum, Pt_1(3)/CNT (and Pt_1(1)/CNT and Pt_1(0.15)/CNT) exhibited an almost identical XRD pattern to that of a Pt-free CNT-supporting substrate (Supplementary Fig. 2). More evidently, the k^3-weighted Pt L_3-edge extended X-ray absorption fine structure (EXAFS) spectra revealed a strong scattering peak at approximately 2.0 Å (Supplementary Fig. 3), which corresponds to Pt–N bonding. Their fitting parameters further confirmed the first-shell Pt–N bonding and second-shell Pt–C bonding without any Pt–Pt bonding. The coordination number (CN) of the Pt–N bonding for Pt_1(3)/CNT was approximately four (Supplementary Table 2). Both the X-ray absorption near edge structure (XANES) and X-ray photoelectron spectroscopy (XPS) spectra verified that the oxidation state of the Pt species on Pt_1(3)/CNT was Pt^{II} (Supplementary Figs. 4 and 5). Therefore, these physical characterization results revealed that Pt_1(3)/CNT was composed of abundant porphyrin-like Pt^{II}–N_4 moieties covalently embedded on the CNT support.

Supplementary Note 2

Non-Faradaic O_2 formation during online DEMS measurements

For product analysis, online differential electrochemical mass spectrometry (DEMS) measurement was conducted for $Pt_1(X)$/CNTs (X = 0.15, 1, and 3), which enables a potential-resolved quantification of gaseous or volatile products in situ. The ionic currents for *m*/*z* = 32 and 35 were recorded to identify the main products of oxygen evolution reaction (OER) and chlorine evolution reaction (CER), i.e., O_2 and Cl_2, during two slow cyclic voltammograms (CVs). In addition, we further take into account the possible Cl_2 hydrolysis before introducing into the mass spectrometer (including an electrolyte, a porous polytetrafluoroethylene (PTFE) membrane, and a capillary connecting the electrochemical flow cell and mass spectrometer) according to the following equation: $Cl_2 + H_2O \rightarrow HCl + HOCl$[1,2]. Thus, the ionic currents for *m*/*z* = 36 and 51 were additionally monitored for HCl and HOCl, respectively.

For all $Pt_1(X)$/CNTs, the online DEMS results reveal a predominant ionic current for *m*/*z* = 35 (Cl^+) in 0.1 M $HClO_4$ + 1 M NaCl electrolyte, which corresponds to the fragmentation of Cl_2 and its hydrolyzed derivatives, i.e., HCl and HOCl (Fig. 1 and Supplementary Fig. 14). Concurrently, distinct ionic currents for *m*/*z* = 36 and 51 (HCl^+ and OCl^+, respectively) are detected, indicating the formation of HCl and HOCl during CER. An insignificant ionic current for *m*/*z* = 32 (O_2^+) is identified, corresponding to the molecular ion of O_2, although the $Pt_1(X)$/CNTs exhibited almost 100% selectivity toward CER based on the rotating ring disk electrode (RRDE) measurements (Supplementary Fig. 6).

This discrepancy possibly originates from the further HOCl decomposition in the vacuum system, forming O_2 as a product by the following equation: $2HOCl \rightarrow 2HCl + O_2$[1,2]. To confirm the aforementioned scenario for O_2 formation, we further performed an identical experiment in the absence of NaCl in the electrolyte. Contrasting with the results obtained in the presence of NaCl, the DEMS results reveal no detectable signals for O_2 and Cl_2 formation during CVs (Fig. 1c). Given that 1) O_2 formation does not occur without Cl_2 evolution, and 2) the potential range for O_2 formation closely matches those of Cl_2 and HOCl formations, we can reasonably conclude that the O_2 signal during CER, as shown in Fig. 1b and Supplementary Fig. 14, is not a result of Faradaic reaction, i.e., OER, but an artifact resulting from the Cl_2 hydrolysis and subsequent HOCl decomposition in the vacuum system of our DEMS setup.

Supplementary Note 3

DFT calculations

1. Computational details

Electronic structure calculations for periodically replicated appropriate models were performed using the Vienna ab initio simulation package (VASP 5.4.1) based on the framework of density functional theory (DFT)[3]. The exchange–correlation potential was treated as in the generalized gradient approximation (GGA) with form proposed by Perdew–Burke–Ernzerhof (PBE)[4]. The valence electron density was expanded on a plane wave basis set with an optimal kinetic energy cutoff of 415 eV since test calculations for the *, *Cl, *O, and *OCl adsorbates with a kinetic energy cutoff of 600 eV indicate that the total energies are affected by at most 0.01 eV. The projected augmented wave (PAW) method[5], as implemented in VASP by Kresse and Joubert[6], was used to take into account the effect of core electrons on the valence electron density. To model the $Pt–N_x$ sites, graphene-like patch cluster models were used with two carbon rings surrounding the $Pt–N_x$ active sites, capped with H-bonds. This avoids using an exceedingly large supercell in a periodic approach necessary to avoid the long-range distortion on graphene induced by the $Pt–N_x$ sites while large enough to lead to converged results. To carry out the necessary numerical integrations in the reciprocal space, the Brillouin zone was sampled using a 4×4×1 **k**-point **Γ**-centered Monkhorst-Pack grid. Using the **Γ**-point only results in a difference of the total energy of at most 0.01 eV. A convergence criterion of 10^{-5} eV was used for the total energy, while the relaxation of atomic positions was stopped when forces acting on all relaxed atoms were smaller than 0.01 eV $Å^{-1}$. The calculation of vibrational frequencies for the optimized geometries was carried out by taking the elements of the Hessian matrix as finite differences of analytical gradients with intervals of 0.03 Å. A vacuum width of 25 Å was added to the *x*- and *y*-direction (along the plane direction defined by the employed models), whereas a vacuum width of 20 Å was added in the *z*-direction in all cases to avert artificial interactions between the periodically repeated models. An effective description of the dispersion interactions was included using the Grimme's DFT-D3 method[7]. The adsorption energy was calculated as follows:

$$E_{ads} = E_{i/sub} - (E_{sub} + E_i) \quad (1),$$

where E_{sub} is the energy of relaxed $Pt–N_4$ or $Pt–N_3(V)$, E_i is the energy of the reference molecule, and $E_{i/sub}$ is the energy of the intermediate adsorbed on the active Pt sites of $Pt–N_4$ or $Pt–N_3(V)$.

Based on this definition, it follows the more negative the E_{ads} value, the more stable the adsorption structure.

The CER/OER performance was evaluated by calculating the free-energy changes (ΔG) for each elementary reaction step according to the following equation:

$$\Delta G = \Delta E + \Delta E_{ZPE} - T\Delta S \qquad (2),$$

where ΔE corresponds to E_{ads} (cf. Equation (1)), ΔE_{ZPE} is the change in zero-point energy for the step of interest, T is the temperature in Kelvin, and ΔS is the change in entropy. ΔE_{ZPE} was obtained directly from the calculated vibrational frequencies in the harmonic approximation, whereas the $T\Delta S$ term requires evaluating the vibrational partition functions, which are also related to the vibrational frequencies[8].

2. Surface Pourbaix diagrams

In this study, the free energies of the intermediate structures (*Cl, *OCl, *O, *OH, and *OOH) were considered for Pt–N_4 and Pt–N_3(V). To include the applied electrode potential, U, in the analysis of free-energy changes, the computational hydrogen electrode approach (CHE) was used[9]. This was achieved by considering the stoichiometric coefficients of the transferred electrons (e^-) and protons (H^+), denoted as $\nu(e^-)$ and $\nu(H^+)$, respectively, when compiling reaction equations for each adsorption process[10]. We derive the following formula:

$$\Delta G\,(\mathrm{pH}, U) = \Delta E_{tot} + \Delta E_{ZPE} - T\Delta S - \nu(H^+)\ 0.059\ \mathrm{pH} - \nu(e^-) \cdot eU \qquad (3).$$

The value of 0.059 eV was derived from the term $k_BT \cdot \ln 10$ evaluated at room temperature, where k_B is Boltzmann's constant and U is the applied electrode potential on the standard hydrogen electrode (SHE) scale[11].

The most thermodynamically favorable structure was determined by the minimization of the ΔG values for each adsorbate among the set of considered surface structures. The resulting surface phase (Pourbaix) diagram is shown as a function of overpotential η, defined by $\eta = U - 1.36$ V. For the analysis, the pH was fixed at zero, because we aimed to comprehend trends for the anodic CER in an acidic medium[12]. Hence, we are not discussing pH effects for which the application of grand canonical schemes is called for[13].

3. Mechanistic studies: Assessment of electrocatalytic activity

Free-energy diagrams for the CER over Pt–N_4 and Pt–N_3(V) were constructed to evaluate the

CER activity of these active sites. Based on the knowledge gained in previous studies[14-16], the CER was assumed to proceed via the Volmer–Heyrovsky mechanism[17,18]. Two different Volmer–Heyrovsky pathways with dissimilar intermediates (i.e., *Cl or *OCl) were considered in our theoretical study[19].

(I) Pathway mediated by the *Cl intermediate:

ΔG_1: $* + Cl^-(aq) \rightleftharpoons *Cl + e^-$ (4),

ΔG_2: $*Cl + Cl^-(aq) \rightleftharpoons Cl_2(g) + e^-$ (5).

(II) Pathway mediated by the *OCl intermediate:

ΔG_3: $*O + Cl^-(aq) \rightleftharpoons *OCl + e^-$ (6),

ΔG_4: $*OCl + Cl^-(aq) \rightleftharpoons *O + Cl_2(g) + e^-$ (7).

The free energy of chloride in solution, $G\,(Cl^-_{aq})$, is related to that of the Cl_2 gas molecule as follows:

$Cl^-(aq) \rightleftharpoons 1/2Cl_2(g) + e^-$, $\Delta G = 0$ eV @ $U = 1.36$ V_{SHE} (8).

Consequently, the ΔG_j values for each elementary step are given by:

$\Delta G_1 = -\Delta G_2 = G\,(*Cl) - 1/2 \cdot G\,(Cl_2) - G\,(*)$ (9),

$\Delta G_3 = -\Delta G_4 = G\,(*OCl) - 1/2 \cdot G\,(Cl_2) - G\,(*O)$ (10),

where G (*Cl), G (*O), and G (*OCl) are the total energies of the adsorbed intermediates and G (*) is the total energy of Pt–N_4 and Pt–N_3(V) without adsorbed intermediates. Note that the relations $\Delta G_1 = -\Delta G_2$ and $\Delta G_3 = -\Delta G_4$ are fulfilled at the CER equilibrium potential, that is, $U = 1.36$ V_{SHE}.

The OER, $2H_2O(l) \rightarrow O_2(g) + 4H^+(aq) + 4e^-$, $U°_{OER} = 1.23$ V vs. reversible hydrogen electrode (RHE), was modelled by assuming the mononuclear mechanism[20]:

ΔG_5: $* + H_2O(l) \rightleftharpoons *OH + H^+ + e^-$ (11),

ΔG_6: $*OH \rightleftharpoons *O + H^+ + e^-$ (12),

ΔG_7: $*O + H_2O(l) \rightleftharpoons *OOH + H^+ + e^-$ (13),

ΔG_8: $*OOH \rightleftharpoons * + O_2(g) + H^+ + e^-$ (14).

The free energy of a proton-electron pair, G ($H^+ + e^-$), can be related to the H_2 gas molecule using the CHE approach as follows:

$$H^+ + e^- \rightleftharpoons 1/2H_2\text{ (g)}, \Delta G = 0\text{ eV @ } U = 0\text{ V}_{SHE} \quad (15).$$

Therefore, the ΔG values for each elementary step are:

$$\Delta G_5 = G\,(*OH) + 1/2 \cdot G\,(H_2) - G\,(H_2O) - G\,(*) \quad (16),$$

$$\Delta G_6 = G\,(*O) + 1/2 \cdot G\,(H_2) - G\,(*OH) \quad (17),$$

$$\Delta G_7 = G\,(*OOH) + 1/2 \cdot G\,(H_2) - G\,(H_2O) - G\,(*O) \quad (18),$$

$$\Delta G_8 = 4 \times 1.23\text{ eV} - (\Delta G_5 + \Delta G_6 + \Delta G_7) \quad (19),$$

where G (*OH), G (*O), and G (*OOH) are the total energies of the adsorbed intermediates, and G (*) is the total energy of Pt–N_4 and Pt–N_3(V) without adsorbed intermediates.

To quantify the electrocatalytic activity for the CER and OER, the recently introduced descriptor G_{max} (U), which is an activity measure that goes beyond the conventional approach in terms of the thermodynamic overpotential only, was used[21,22]. This descriptor relies on a free-energy span model by extracting the largest free-energy difference between intermediate states at a given target electrode potential:

$$G_{max}\,(U) = \max[G_{span\,\#k}\,(U), k = 1, \ldots, n] \quad (20).$$

For further information on how to define the free-energy spans, $G_{span\,\#k}$ (U), for a two-electron (CER) or four-electron process (OER), we refer the reader to recent publications by the authors[23,24].

4. Selectivity assessment

The descriptor G_{max} (U) was extracted for both OER and CER at well-defined electrode potentials, U >1.36 V_{SHE}. The free-energy difference, G_{sel} (U), defined as

$$G_{sel}\,(U) = G_{max}\,(U)^{OER} - G_{max}\,(U)^{CER} \quad (21),$$

is a measure of the CER selectivity[25]. This quantity was used to determine the CER selectivity in percentage using the following relation[19,25]:

$$\text{CER selectivity } (U) = \frac{\exp\left(\frac{G_{sel}}{k_B.T}\right)}{\exp\left(\frac{G_{sel}}{k_B.T}\right)+1} \quad (22).$$

5. Stability assessment

Considering that PtO_2 is the preferred state of Pt under CER conditions[26], the tendency of the active Pt^{II} sites toward oxidative demetallation to PtO_2 was analyzed for Pt–N_4 and Pt–N_3(V). More precisely, the free-energy change, ΔG_{stab}, for the equation [Pt] + $2H_2O$ → [ ] + PtO_2 + $4H^+ + 4e^-$ was determined by DFT. Here, [ ] denotes the empty pocket of the Pt–N_4 and Pt–N_3(V) moieties. Based on the obtained ΔG_{stab} value, the equilibrium dissolution potential, U_{diss}, was calculated as follows:

$$U_{diss} = \frac{\Delta G_{stab}}{4 \cdot e} \quad (23).$$

The U_{diss} value indicates the electrode potential at which the oxidative demetallation of central Pt species to PtO_2 becomes thermodynamically favorable. Adequate stability of the Pt–N_4 and Pt–N_3(V) sites is provided if the U_{diss} value significantly exceeds the CER equilibrium potential (i.e., 1.36 V_{SHE}).

We note that the U_{diss} value cannot be used to explain why Pt–N_3(V) is first formed when the Pt loading is low. We believe that the formation of Pt–N_3(V) for low Pt loadings is governed by a kinetic reaction control, whereas with higher Pt loadings, the formation of Pt–N_4 prevails since this configuration is thermodynamically preferred (thermodynamic reaction control), as evident from its larger U_{diss} value.

Supplementary Figures and Tables

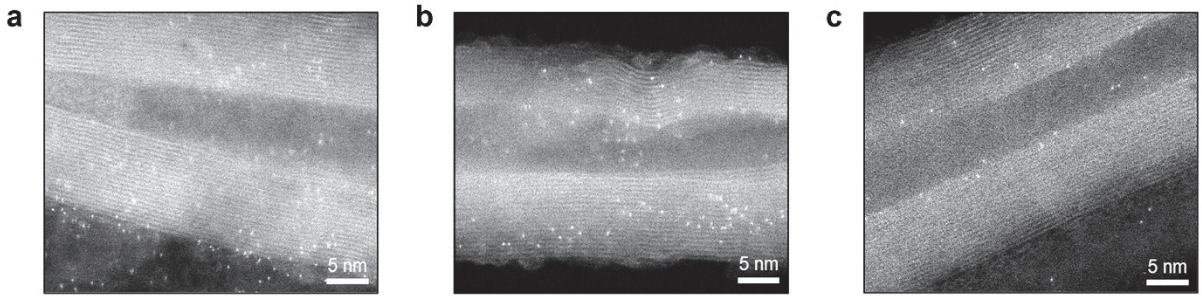

Supplementary Fig. 1: a–c HAADF-STEM images of Pt_1(3)/CNT (**a**), Pt_1(1)/CNT (**b**), and Pt_1(0.15)/CNT (**c**).

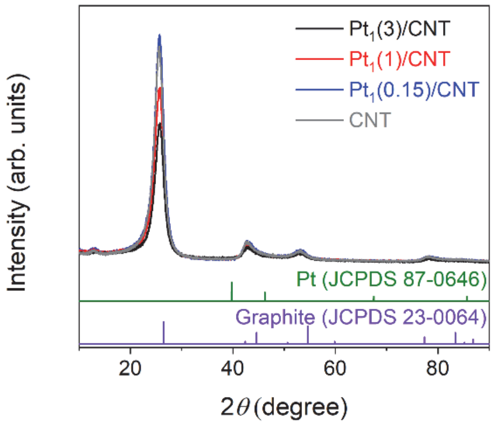

Supplementary Fig. 2: XRD patterns of the Pt_1(X)/CNTs and CNT supporting substrate. For better comparison, XRD patterns of graphite (JCPDS 23-0064) and Pt (JCPDS 87-0646) are also shown.

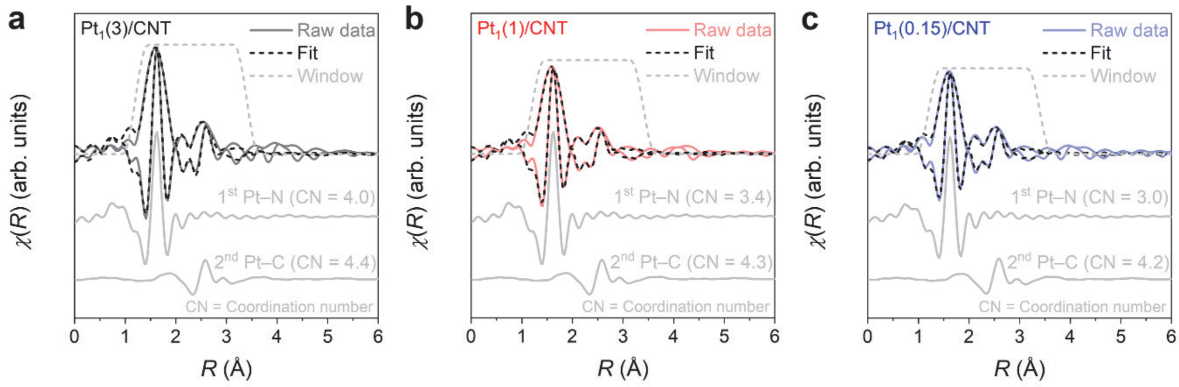

Supplementary Fig. 3: a–c The k^3-weighted Pt L_3-edge EXAFS spectra and fitted curves of Pt_1(3)/CNT (**a**), Pt_1(1)/CNT (**b**), and Pt_1(0.15)/CNT (**c**). The detailed fitting parameters are provided in Supplementary Table 2.

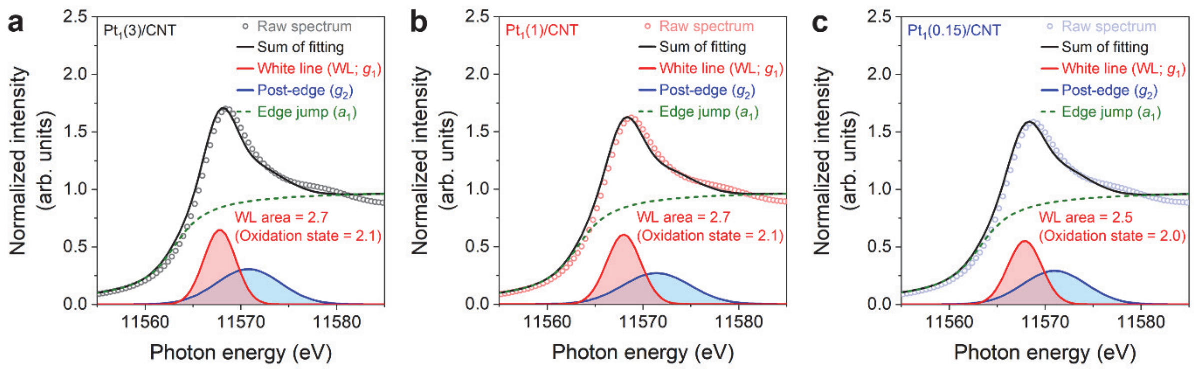

Supplementary Fig. 4: a–c Pt L_3-edge XANES spectra of Pt_1(3)/CNT (**a**), Pt_1(1)/CNT (**b**), and Pt_1(0.15)/CNT (**c**). The results show that the average oxidation state of the catalysts is Pt^{II}. The XANES white line (WL) fitting was conducted using the interpolation equation of Pt references in our previous report[15]. The fitting consists of one arctangent function ($a_1(E)$) and two Gaussian functions ($g_1(E)$ and $g_2(E)$) by the following equation: $I(E) = a_1(E) + g_1(E) + g_2(E)$.

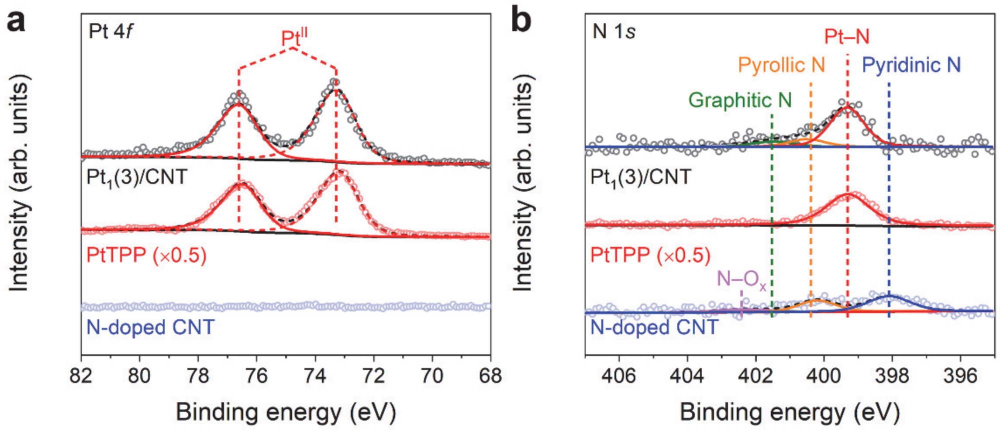

Supplementary Fig. 5: a XPS Pt 4*f* and **b** N 1*s* spectra of $Pt_1(3)$/CNT, PtTPP, and N-doped CNT. The XPS Pt $4f_{7/2}$ spectrum of $Pt_1(3)$/CNT shows strong peaks at 73.2 and 76.6 eV, almost identical to those of PtTPP with a Pt^{II} center.

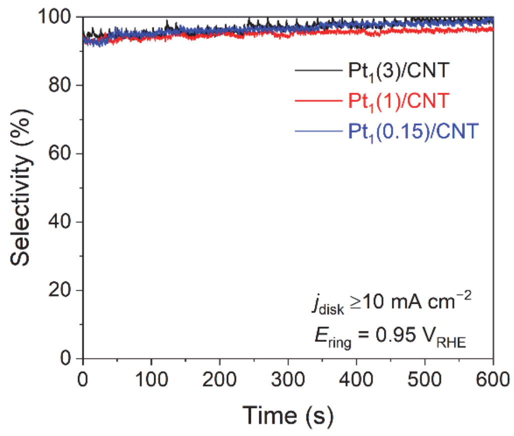

Supplementary Fig. 6: CER selectivity of Pt_1(3)/CNT, Pt_1(1)/CNT, and Pt_1(0.15)/CNT measured using an RRDE in Ar-saturated 0.1 M $HClO_4$ with 1 M NaCl. The CER selectivity of all the catalysts is almost 100%.

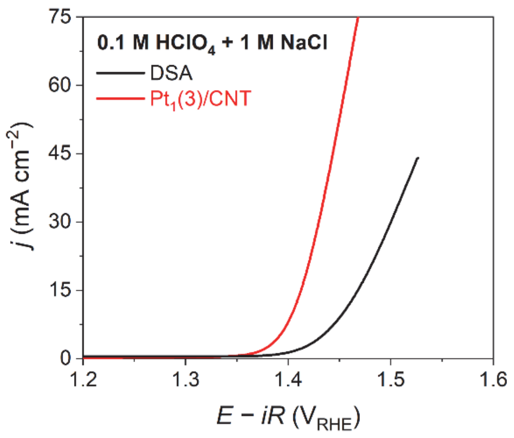

Supplementary Fig. 7: CER polarization curves of Pt_1(3)/CNT and Ru/Ir-based dimensionally stable anode (DSA; Ru/Ir atomic ratio = 0.5; provided by Siontech Inc.). The Pt_1(3)/CNT has a much higher CER activity than DSA.

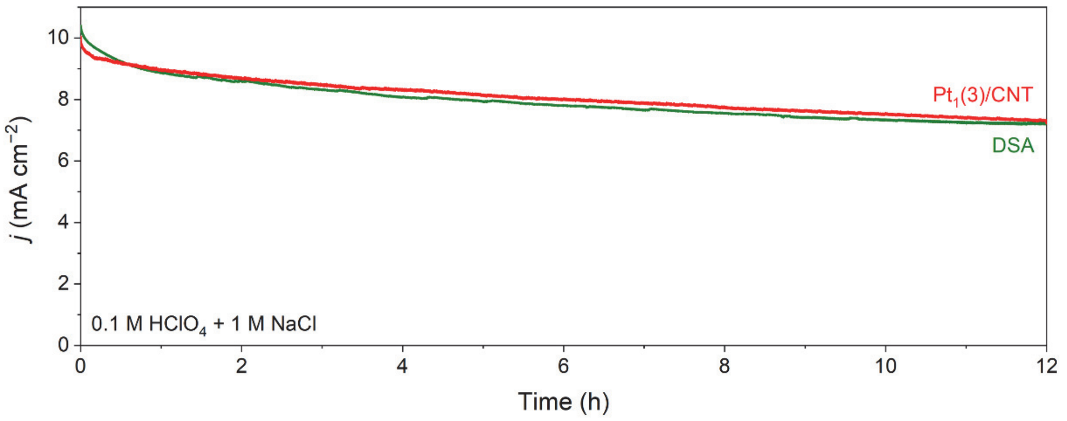

Supplementary Fig. 8: Durability tests for the Pt_1(3)/CNT and commercialized DSA electrode under constant potential at an initial current density of 10 mA cm^{-2}.

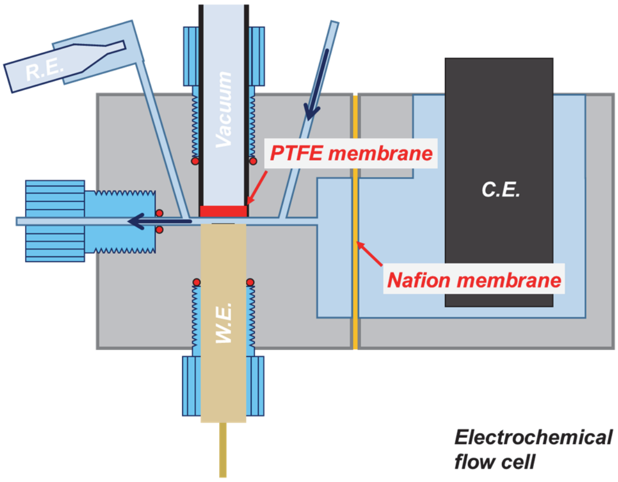

Supplementary Fig. 9: Schematic image of a homemade electrochemical flow cell (EFC) connected to the inductively coupled plasma-mass spectrometry (ICP-MS). A PTFE membrane-sealed Teflon tube was installed into the EFC to remove the gaseous chlorine product by vacuum. The Nafion membrane was used to separate the counter electrode from the main body of the EFC.

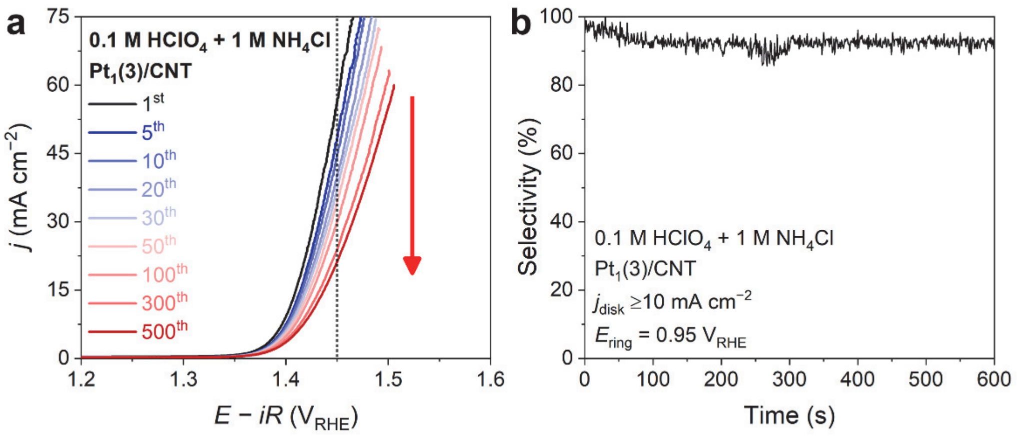

Supplementary Fig. 10: **a** CER activity and durability measurements in Ar-saturated 0.1 M $HClO_4$ with 1 M NH_4Cl. **b** CER selectivity of Pt_1(3)/CNT measured using an RRDE in Ar-saturated 0.1 M $HClO_4$ with 1 M NH_4Cl. The CER activity and selectivity of Pt_1(3)/CNT are comparable with those measured in Ar-saturated 0.1 M $HClO_4$ with 1 M NaCl, indicating no significant effects of the Cl^- precursors on the CER electrocatalysis on Pt_1(3)/CNT. Therefore, to prevent the harmful accumulation of NaCl on the sampler and skimmer cones of the ICP-MS instrument, NH_4Cl was used as a Cl^- precursor for the online EFC/ICP-MS studies.

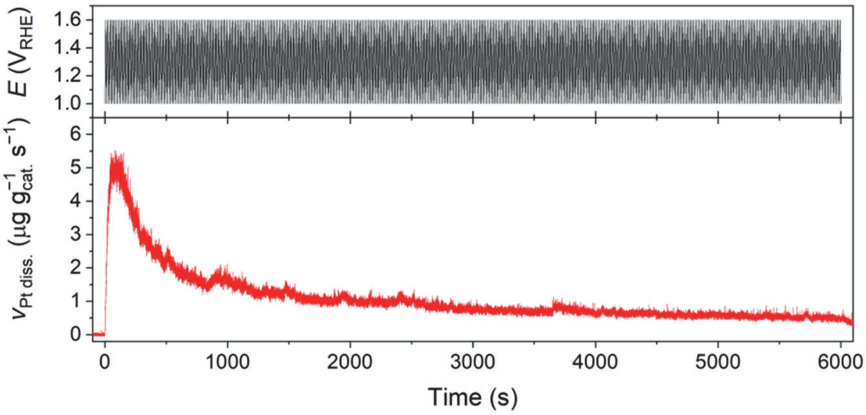

Supplementary Fig. 11: Real-time Pt dissolution of $Pt_1(3)$/CNT. The online EFC/ICP-MS signal measured during 500 CV cycles in the potential range of 1.0–1.6 V_{RHE} in Ar-saturated 0.1 M $HClO_4$ with 1 M NH_4Cl.

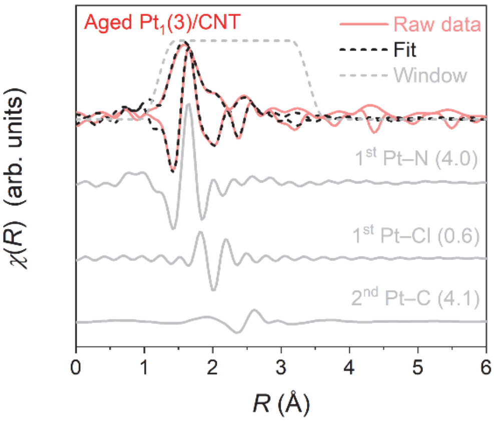

Supplementary Fig. 12: The k^3-weighted Pt L_3-edge EXAFS spectrum and fitted curves of $Pt_1(3)$/CNT after 500 CVs in the potential range of 1.0–1.6 V_{RHE} in Ar-saturated 0.1 M $HClO_4$ with 1 M NaCl. The detailed fitting parameters are provided in Supplementary Table 2.

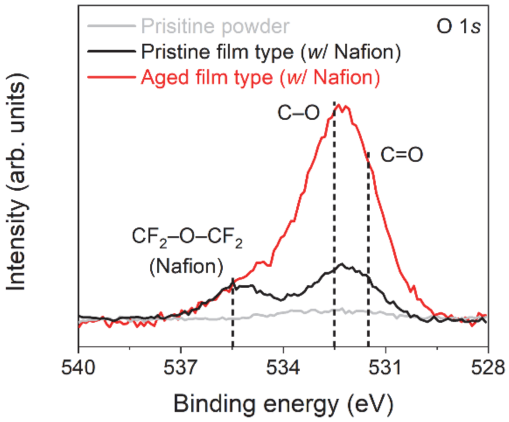

Supplementary Fig. 13: The XPS O 1*s* spectra of pristine Pt_1(3)/CNT powder sample, pristine Pt_1(3)/CNT film with Nafion binder, and aged Pt_1(3)/CNT film with Nafion binder after 500 CVs in the potential range of 1.0–1.6 V_{RHE} in Ar-saturated 0.1 M $HClO_4$ with 1 M NaCl. Fabrication of powder Pt_1(3)/CNT with the Nafion ionomer is a prerequisite for preparing the working electrode. Consequently, the XPS O 1*s* spectrum of the aged Pt_1(3)/CNT film results from convoluted signals of the newly generated oxygen functionalities and Nafion ionomer[27,28]. Thus, the XPS O 1*s* spectrum of the pristine Pt_1(3)/CNT film was subtracted from that of the aged Pt_1(3)/CNT film to deconvolute the XPS signals of the newly generated oxygen functionalities without the Nafion contribution (shown in Fig. 2a in the main article).

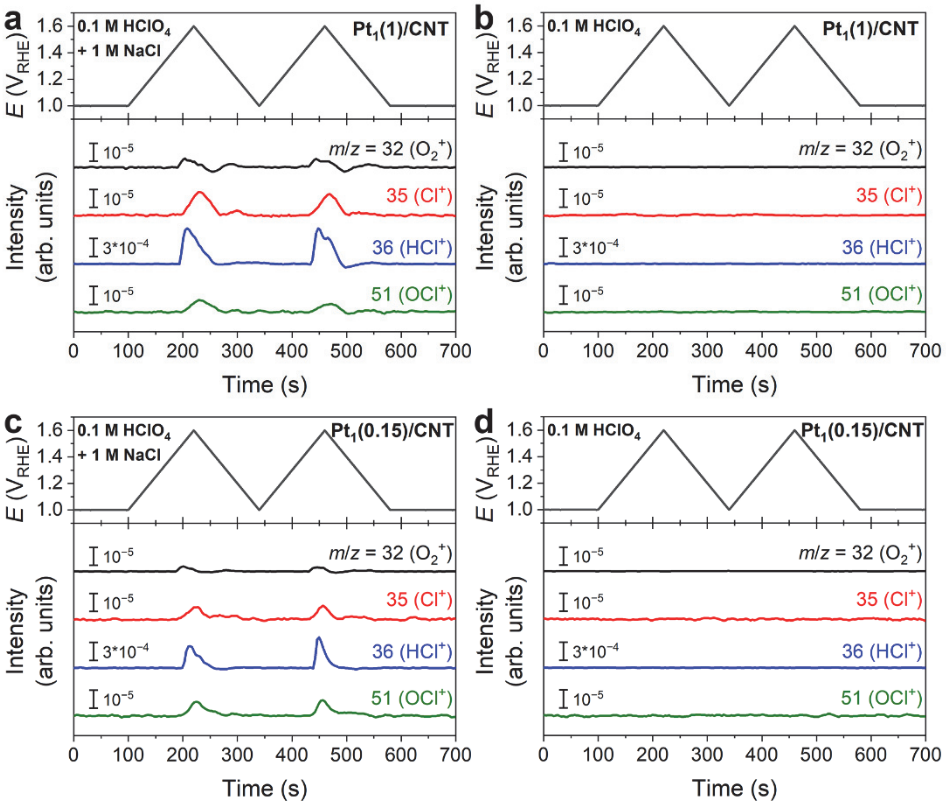

Supplementary Fig. 14: a–d Online DEMS results of *m*/*z* = 32, 35, 36, and 51 of Pt_1(1)/CNT (**a,b**) and Pt_1(0.15)/CNT (**c,d**) during two consecutive slow CVs obtained in Ar-saturated 0.1 M $HClO_4$ with 1 M NaCl (**a,c**) and in NaCl-free electrolytes (**b,d**).

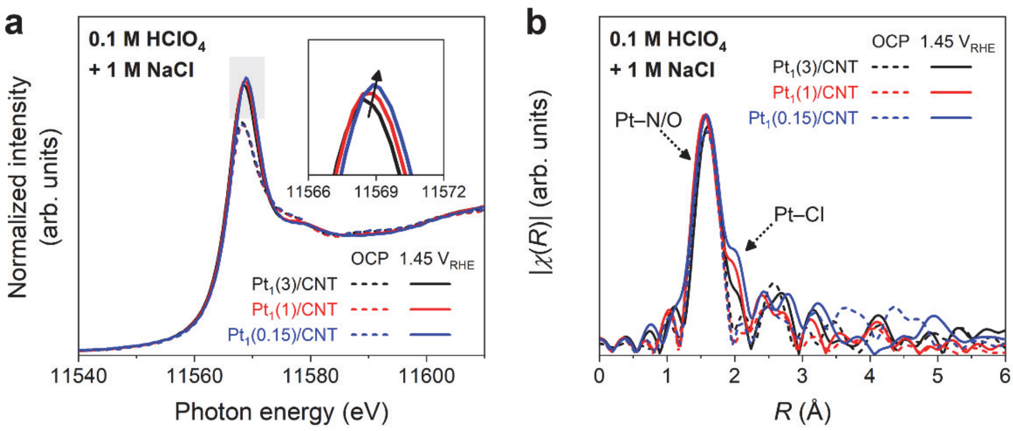

Supplementary Fig. 15: a The Pt L_3-edge in situ XANES spectra of Pt_1(X)/CNTs (X = 0.15, 1, and 3) measured in Ar-saturated 0.1 M $HClO_4$ + 1 M NaCl electrolyte at open-circuit potential (OCP) and 1.45 V_{RHE}. **b** The k^3-weighted Pt L_3-edge in situ EXAFS spectra of Pt_1(X)/CNTs (X = 0.15, 1, and 3) measured in Ar-saturated 0.1 M $HClO_4$ + 1 M NaCl electrolyte. The detailed EXAFS fitting parameters are provided in Supplementary Table 3.

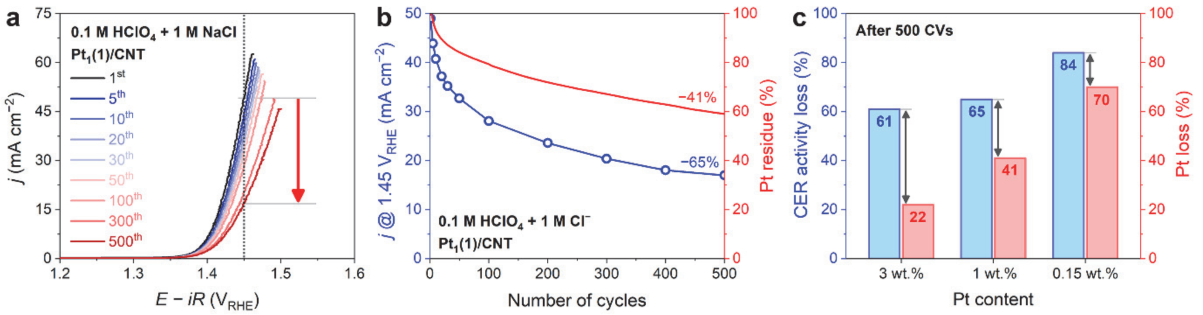

Supplementary Fig. 16: a,b Comparison of the CER activity decrement of Pt_1(1)/CNT (**a**) and Pt loss measured by online EFC/ICP-MS (**b**) during durability test. **c** A summary of CER activity and Pt losses of Pt_1(X)/CNTs (X = 0.15, 1, and 3) after durability test.

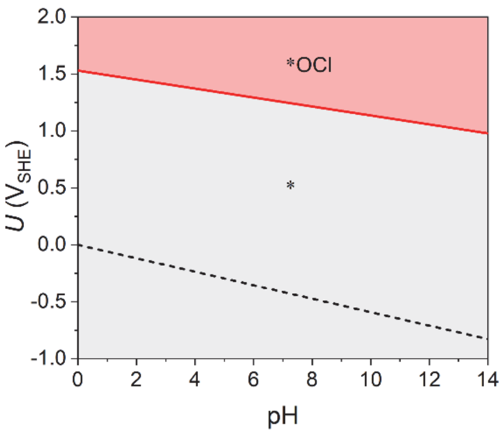

Supplementary Fig. 17: Surface Pourbaix diagram of the square planar Pt–N_4 model.

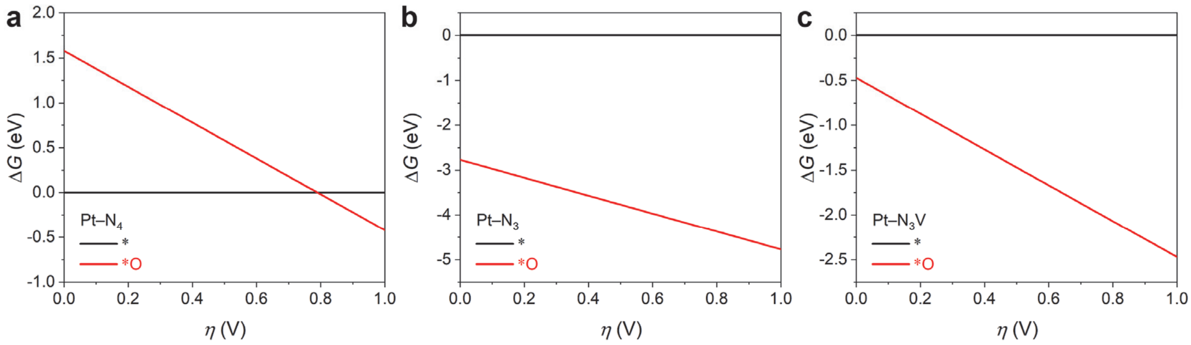

Supplementary Fig. 18: Pourbaix-like diagrams for the **a** square planar Pt–N_4, **b** trigonal planar Pt–N_3, and **c** T-shaped Pt–N_3V models.

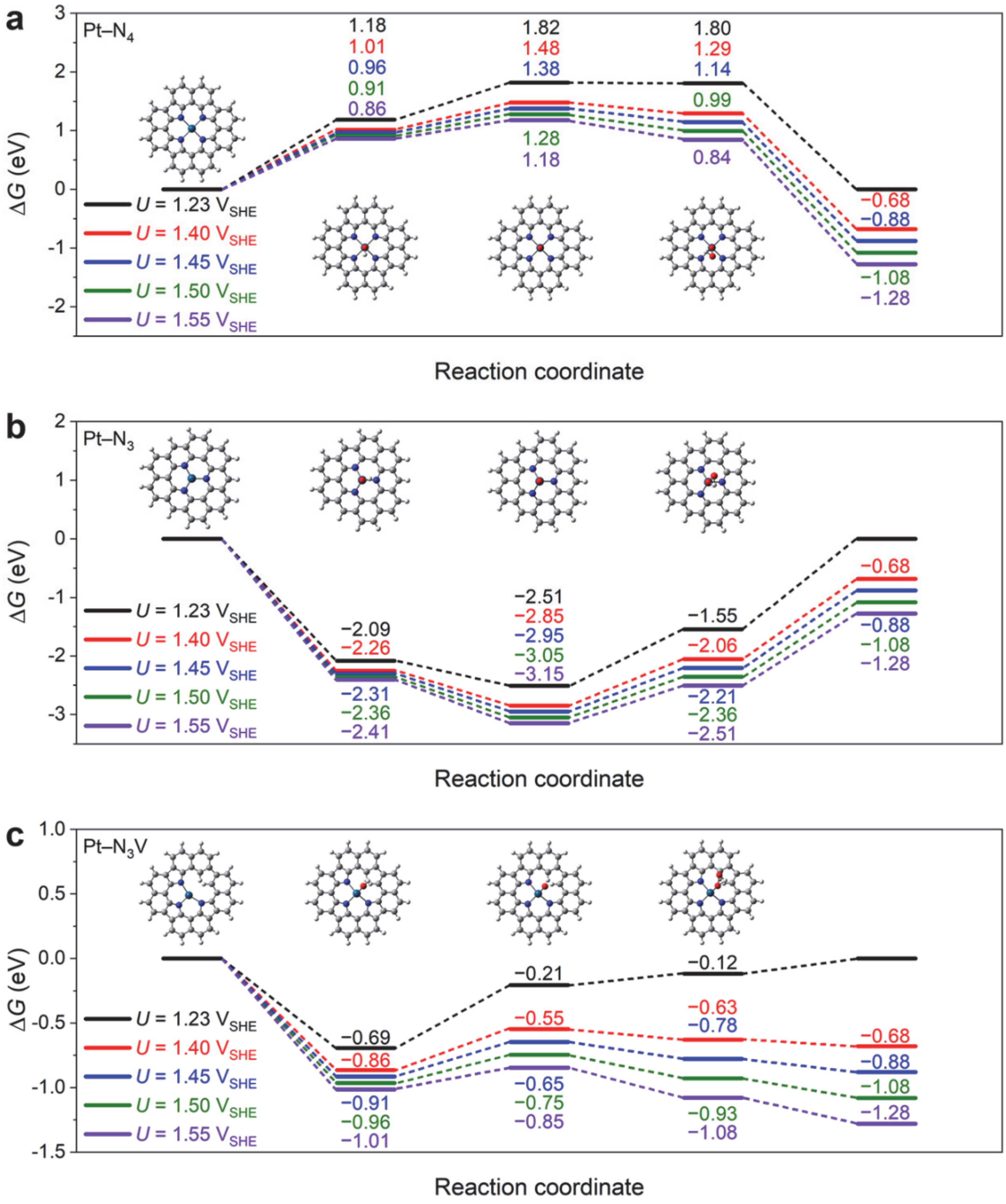

Supplementary Fig. 19: a–c Free-energy diagrams of the OER, assuming the mononuclear mechanism via the *OH, *O, and *OOH adsorbates, for Pt–N$_4$ (**a**), Pt–N$_3$ (**b**), and Pt–N$_3$V (**c**).

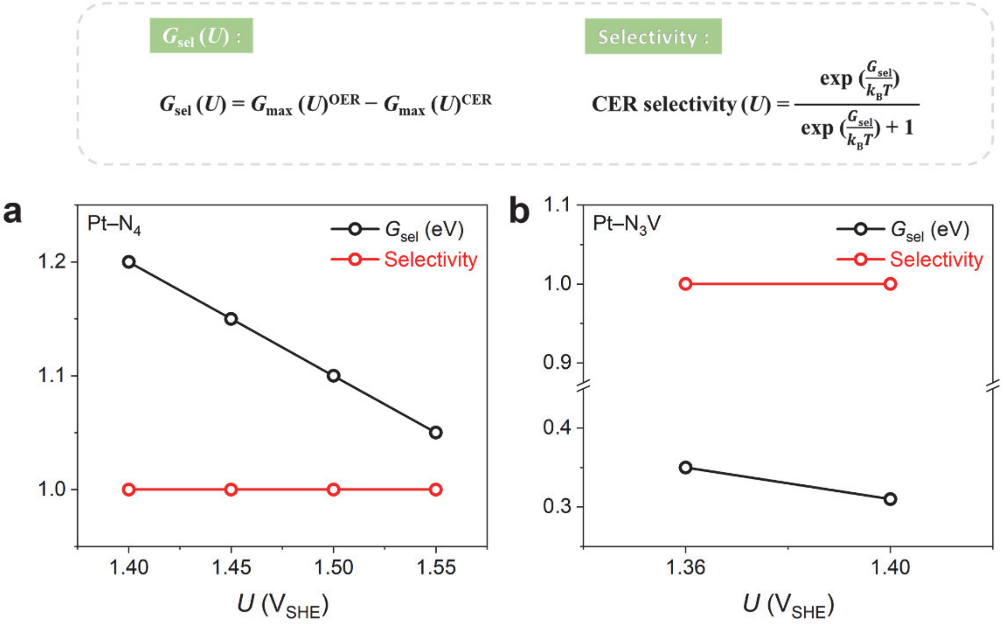

Supplementary Fig. 20: Selectivity analysis for the competing CER and OER processes over the **a** Pt–N_4 and **b** Pt–N_3V sites.

Supplementary Table 1: Comparison of CER activities and operational conditions for previously reported CER catalysts in acidic media.

**Catalysts**	**η @ 10 mA cm^{-2} (mV)**	**CER operation conditions**	**Precious metal contents**	**Ref.**
$Pt_1(3)/CNT$	50	0.1 M $HClO_4$ + 1 M NaCl (25 °C)	3wt.% Pt	This work
Commercial DSA, Ru−Ti−Ir/Ti (Siontech, Korea)	105	0.1 M $HClO_4$ + 1 M NaCl (25 °C)	NA	This work
RuO_2 (110)	140	0.1 M $HClO_4$ + 1 M NaCl (25 °C)	76wt.% Ru	29
Commercial DSA, Ru−Ti−Ir/Ti (Covestro, Germany)	90	3.0 M $NaNO_3$ + 1 M NaCl (pH 3 adjusted by adding HCl, 25 °C)	NA	30
$Ru_{0.3}Sn_{0.7}O_2/Ti$	32	3.5 M NaCl (pH 3, 80 °C)	21wt.% Ru	31
Mesoporous Ru−Ir/TiO_2	140	4.0 M NaCl (pH 3, 40 °C)	7.5wt.% Ru 7.5wt.% Ir	32
Ir_1-TiC	30	Cl_2-saturated 4 M NaCl (pH 2 adjusted by adding HCl, 25 °C)	0.41wt.% Ir	33
$RuTiO_x$	130	Cl_2-saturated 4 M NaCl (pH 2 adjusted by adding HCl, 25 °C)	NA	34
$NiSb_2O_x$	410	Cl_2-saturated 4 M NaCl (pH 2 adjusted by adding HCl, 25 °C)	NA	34

Supplementary Table 2. Summary of EXAFS fitting parameters of pristine Pt_1(X)/CNTs, aged Pt_1(3)/CNT, and Pt foil.

**Sample**	***k* range**	***R* range**	**Shell**	**CN**	***R* (Å)**	**σ^2 (10^{-3} Å^{-2})**	**ΔE_0 (eV)**	***R*-factor (%)**
Pt_1(0.15)/CNT	2.7−11.2	1.2−3.4	Pt−N	3.0 (± 0.3)	2.01 (± 0.01)	3.56 (± 0.97)	16.43 (± 0.33)	1.2
			Pt···C	4.2 (± 1.3)	2.99 (± 0.02)	8.44 (± 4.01)		
Pt_1(1)/CNT			Pt−N	3.4 (± 0.4)	2.00 (± 0.01)	4.26 (± 1.00)	15.59 (± 0.91)	1.2
			Pt···C	4.3 (± 1.5)	2.97 (± 0.02)	9.38 (± 4.71)		
Pt_1(3)/CNT			Pt−N	4.0 (± 0.4)	2.01 (± 0.01)	3.58 (± 0.99)	15.89 (± 1.00)	1.3
			Pt···C	4.4 (± 1.6)	3.01 (± 0.00)	6.42 (± 4.11)		
Aged Pt_1(3)/CNT	2.5−11.2	1.2−3.4	Pt−N	4.0 (± 0.5)	2.02 (± 0.01)	4.12 (± 1.37)	14.37 (± 1.41)	1.3
			Pt−Cl	0.6 (± 0.1)	2.34 (± 0.02)	1.00*		
			Pt···C	4.1 (± 0.8)	2.99 (± 0.04)	10.64 (± 5.49)		
Pt foil	2.1−13.5	1.7−3.8	Pt−Pt	12*	2.77 (± 0.02)	4.59 (± 0.27)	9.26 (± 0.50)	0.3

Pt−N indicates a single scattering path of the first-shell. Pt···C indicates a single scattering path of the second-shell (Shell column). The CN is the coordination number obtained from the amplitude reduction factor (S_0^2) of 0.84. *R* indicates bond distance. σ^2 indicates the Debye-Waller factor. ΔE_0 indicates the energy shift. *R*-factor was obtained from the best fit for the respective catalysts. (*Defined parameters to reduce correlations between variables)

Supplementary Table 3: Summary of EXAFS fitting parameters of Pt_1(X)/CNTs (X = 0.15, 1, and 3) measured in Ar-saturated 0.1 M $HClO_4$ + 1 M NaCl electrolyte.

**Sample**	***k* range**	***R* range**	**Shell**	**CN**	***R* (Å)**	**σ^2 (10^{-3} Å^{-2})**	**ΔE_0 (eV)**	***R*-factor (%)**
Pt_1(0.15)/CNT @ OCP	2.7−11.2	1.2−2.6	Pt−N/O	3.9 (± 0.7)	1.99 (± 0.01)	3.04 (± 1.48)	10.15 (± 2.21)	1.9
Pt_1(0.15)/CNT @ 1.45 V_{RHE}			Pt−N/O	4.1 (± 0.6)	2.00 (± 0.03)	2.50 (± 1.84)	12.74 (± 3.67)	2.4
			Pt−Cl	1.0 (± 0.3)	2.31 (± 0.03)	1.00*		
Pt_1(1)/CNT @ OCP			Pt−N/O	4.1 (± 0.6)	1.98 (± 0.01)	3.10 (± 1.28)	9.49 (± 2.84)	2.3
Pt_1(1)/CNT @ 1.45 V_{RHE}			Pt−N/O	4.2 (± 0.6)	1.99 (± 0.02)	2.44 (± 1.72)	9.32 (± 3.83)	2.3
			Pt−Cl	0.8 (± 0.3)	2.30 (± 0.04)	1.00*		
Pt_1(3)/CNT @ OCP			Pt−N/O	4.1 (± 0.7)	1.99 (± 0.01)	3.85 (± 1.50)	11.06 (± 2.12)	1.3
Pt_1(3)/CNT @ 1.45 V_{RHE}			Pt−N/O	4.2 (± 0.6)	2.03 (± 0.02)	3.54 (± 1.84)	12.65 (± 3.55)	2.2
			Pt−Cl	0.5 (± 0.3)	2.33 (± 0.04)	1.00*		

Pt−N/O indicates a single scattering path of the first-shell. Pt···C scattering path of the second-shell is excluded to minimize correlations between multiple variables. The CN is the coordination number obtained from the amplitude reduction factor (S_0^2) of 0.84. *R* indicates bond distance. σ^2 indicates the Debye-Waller factor. ΔE_0 indicates the energy shift. *R*-factor was obtained from the best fit for the respective catalysts. (*Defined parameters to reduce correlations between variables)

Supplementary Table 4: Summary of G_{max} (U) for the CER and OER over Pt–N_4 and Pt–N_3 (V) at pH = 0.

G_{max} (U) (eV)	Pt–N_4	Pt–N_3	Pt–N_3V
CER	0.32[a] (0.28[b])	0.21 (0.17)	0.05 (0.01)
OER	1.56 (1.48)	2.25 (2.17)	0.35 (0.31)

[a] U = 1.36 V_{SHE}

[b] U = 1.40 V_{SHE}

SUPPLEMENTARY REFERENCES

1 Karlsson, R. K. B. & Cornell, A. Selectivity between Oxygen and Chlorine Evolution in the Chlor-Alkali and Chlorate Processes. *Chem. Rev.* **116**, 2982−3028 (2016).

2 Macounová, K. M., Simic, N., Ahlberg, E. & Krtil, P. Electrocatalytic Aspects of the Chlorate Process: A Voltammetric and DEMS Comparison of RuO_2 and DSA Anodes. *J. Electrochem. Soc.* **165**, E751 (2018).

3 Li, N. *et al.* Double Transition Metal Carbides MXenes (D-MXenes) as Promising Electrocatalysts for Hydrogen Reduction Reaction: *Ab Initio* Calculations. *ACS Omega* **6**, 23676−23682 (2021).

4 Perdew, J. P. *et al.* Atoms, Molecules, Solids, and Surfaces: Applications of the Generalized Gradient Approximation for Exchange and Correlation. *Phys. Rev. B* **46**, 6671−6687 (1992).

5 Kresse, G. & Furthmüller, J. Efficient Iterative Schemes for *Ab Initio* Total-Energy Calculations using a Plane-Wave Basis Set. *Phys. Rev. B* **54**, 11169−11186 (1996).

6 Kresse, G. & Joubert, D. From Ultrasoft Pseudopotentials to the Projector Augmented-Wave Method. *Phys. Rev. B* **59**, 1758−1775 (1999).

7 Grimme, S. Semiempirical GGA-Type Density Functional Constructed with a Long-Range Dispersion Correction. *J. Comput. Chem.* **27**, 1787−1799 (2006).

8 López, M., Exner, K. S., Viñes, F. & Illas, F. Computational Pourbaix Diagrams for MXenes: A Key Ingredient toward Proper Theoretical Electrocatalytic Studies. *Adv. Theory Simul.*, 2200217 (2022).

9 Nørskov, J. K. *et al.* Origin of the Overpotential for Oxygen Reduction at a Fuel-Cell Cathode. *J. Phys. Chem. B* **108**, 17886−17892 (2004).

10 Exner, K. S., Anton, J., Jacob, T. & Over, H. Chlorine Evolution Reaction on RuO_2(110): *Ab Initio* Atomistic Thermodynamics Study - Pourbaix Diagrams. *Electrochim. Acta* **120**, 460−466 (2014).

11 Kibsgaard, J. *et al.* Designing an Improved Transition Metal Phosphide Catalyst for Hydrogen Evolution using Experimental and Theoretical Trends. *Energy Environ. Sci.* **8**, 3022−3029 (2015).

12 Exner, K. S., Anton, J., Jacob, T. & Over, H. Controlling Selectivity in the Chlorine Evolution Reaction over RuO_2-Based Catalysts. *Angew. Chem. Int. Ed.* **53**, 11032−11035 (2014).

13 Groß, A. Reversible vs Standard Hydrogen Electrode Scale in Interfacial

Electrochemistry from a Theoretician's Atomistic Point of View. *J. Phys. Chem. C* **126**, 11439−11446 (2022).

14 Lim, T. *et al.* Atomically Dispersed Pt–N_4 Sites as Efficient and Selective Electrocatalysts for the Chlorine Evolution Reaction. *Nat. Commun.* **11**, 412 (2020).

15 Lim, T. *et al.* General Efficacy of Atomically Dispersed Pt Catalysts for the Chlorine Evolution Reaction: Potential-Dependent Switching of the Kinetics and Mechanism. *ACS Catal.* **11**, 12232−12246 (2021).

16 Exner, K. S., Anton, J., Jacob, T. & Over, H. Full Kinetics from First Principles of the Chlorine Evolution Reaction over a RuO_2(110) Model Electrode. *Angew. Chem. Int. Ed.* **55**, 7501−7504 (2016).

17 Janssen, L. J. J., Starmans, L. M. C., Visser, J. G. & Barendrecht, E. Mechanism of the Chlorine Evolution on a Ruthenium Oxide/Titanium Oxide Electrode and on a Ruthenium Electrode. *Electrochim. Acta* **22**, 1093−1100 (1977).

18 Trasatti, S. *Electrodes of Conductive Metallic Oxides, Part B (Studies in Physical & Theoretical Chemistry)*. (Elsevier Science Ltd, Amsterdam-Oxford-New York, 1981).

19 Exner, K. S. Design Criteria for the Competing Chlorine and Oxygen Evolution Reactions: Avoid the OCl Adsorbate to Enhance Chlorine Selectivity. *Phys. Chem. Chem. Phys.* **22**, 22451−22458 (2020).

20 Man, I. C. *et al.* Universality in Oxygen Evolution Electrocatalysis on Oxide Surfaces. *ChemCatChem* **3**, 1159−1165 (2011).

21 Exner, K. S. A Universal Descriptor for the Screening of Electrode Materials for Multiple-Electron Processes: Beyond the Thermodynamic Overpotential. *ACS Catal.* **10**, 12607−12617 (2020).

22 Exner, K. S. Why Approximating Electrocatalytic Activity by a Single Free-Energy Change Is Insufficient. *Electrochim. Acta* **375**, 137975 (2021).

23 Razzaq, S. & Exner, K. S. Method to Determine the Bifunctional Index for the Oxygen Electrocatalysis from Theory. *ChemElectroChem* **9**, e202101603 (2022).

24 Exner, K. S. Beyond the Thermodynamic Volcano Picture in the Nitrogen Reduction Reaction over Transition-Metal Oxides: Implications for Materials Screening. *Chin. J. Catal.* **43**, 2871−2880 (2022).

25 Exner, K. S. Controlling Stability and Selectivity in the Competing Chlorine and Oxygen Evolution Reaction over Transition Metal Oxide Electrodes. *ChemElectroChem* **6**, 3401−3409 (2019).

26 Goeke, R. S., Datye, A. K., Atanassov, P. & St-Pierre, J. Model Electrode Structures for Studies of Electrocatalyst Degradation. *ECS Trans.* **33**, 361 (2010).

27 Friedman, A. K., Shi, W., Losovyj, Y., Siedle, A., R. & Baker, L. A. Mapping Microscale Chemical Heterogeneity in Nafion Membranes with X-ray Photoelectron Spectroscopy. *J. Electrochem. Soc.* **165**, H733 (2018).

28 Moulder, J. F. & Chastain, J. *Handbook of X-ray Photoelectron Spectroscopy: A Reference Book of Standard Spectra for Identification and Interpretation of XPS Data*. (Physical Electronics Division, Perkin-Elmer Corporation, 1992).

29 Sohrabnejad-Eskan, I. *et al.* Temperature-Dependent Kinetic Studies of the Chlorine Evolution Reaction over RuO_2(110) Model Electrodes. *ACS Catal.* **7**, 2403−2411 (2017).

30 Zeradjanin, A. R., Menzel, N., Schuhmann, W. & Strasser, P. On the Faradaic Selectivity and the Role of Surface Inhomogeneity during the Chlorine Evolution Reaction on Ternary Ti–Ru–Ir Mixed Metal Oxide Electrocatalysts. *Phys. Chem. Chem. Phys.* **16**, 13741−13747 (2014).

31 Chen, R. *et al.* Microstructural Impact of Anodic Coatings on the Electrochemical Chlorine Evolution Reaction. *Phys. Chem. Chem. Phys.* **14**, 7392−7399 (2012).

32 Menzel, N., Ortel, E., Mette, K., Kraehnert, R. & Strasser, P. Dimensionally Stable Ru/Ir/TiO_2-Anodes with Tailored Mesoporosity for Efficient Electrochemical Chlorine Evolution. *ACS Catal.* **3**, 1324−1333 (2013).

33 Yang, J. *et al.* Regulating the Tip Effect on Single-Atom and Cluster Catalysts: Forming Reversible Oxygen Species with High Efficiency in Chlorine Evolution Reaction. *Angew. Chem. Int. Ed.* **61**, e202200366 (2022).

34 Moreno-Hernandez, I. A., Brunschwig, B. S. & Lewis, N. S. Crystalline Nickel, Cobalt, and Manganese Antimonates as Electrocatalysts for the Chlorine Evolution Reaction. *Energy Environ. Sci.* **12**, 1241−1248 (2019).
